# Independent Component Analysis (ICA) With Covariates Strengthens Behavioral Links in Electroencephalography (EEG) Connectivity

**DOI:** 10.7759/cureus.86533

**Published:** 2025-06-22

**Authors:** Curtis T Cripe, Arnaud Delorme

**Affiliations:** 1 Graduate School of Social Service, Fordham University, New York City, USA; 2 Research, Independent Consultancy, La Jolla, USA

**Keywords:** brain connectivity, electroencephalography (eeg), independent component analysis, quantitative eeg (qeeg), wilcox-johnson tests

## Abstract

The search for reliable biomarkers of clinical and cognitive deficits remains the holy grail of clinical neuroscience, with improved predictive methods and neural correlates paving the way for more effective treatments. Independent component analysis (ICA) has been widely applied in electroencephalography (EEG) signal processing to isolate neural activity from artifacts and noise. This study introduces a novel approach by integrating clinical covariates - behavioral assessments from the Woodcock-Johnson Cognitive Abilities Test III (WJ) Tests - into ICA, enabling the simultaneous decomposition of EEG connectivity patterns and cognitive performance metrics. Using functional connectivity measures as input, we applied two ICA methodologies to a dataset of 175 patients: (1) conventional ICA on EEG connectivity data, followed by correlation analysis with WJ scores, and (2) an augmented ICA approach incorporating both EEG connectivity and WJ measures. Our findings demonstrate that integrating behavioral data into ICA decomposition enhances the significance and robustness of correlations between EEG connectivity and cognitive performance in independent test datasets. These results underscore the potential of ICA with integrated covariates as a powerful multivariate framework for uncovering brain-behavior relationships, offering new insights for clinical and cognitive neuroscience research.

## Introduction

The identification of reliable biomarkers for clinical and cognitive deficits remains a central challenge in neuroscience, as these markers are essential for improving diagnostic accuracy, predicting disease progression, and developing targeted interventions. Advances in neuroimaging and computational techniques have facilitated the discovery of neural correlates underlying cognitive dysfunction, yet robust and generalizable biomarkers remain elusive [1,2]. Electroencephalography (EEG) has emerged as a powerful tool for capturing neural dynamics with high temporal resolution, offering valuable insights into brain function in both healthy and clinical populations. However, the complexity of EEG signals, compounded by artifacts and individual variability, necessitates advanced analytical approaches to extract meaningful biomarkers. Independent component analysis (ICA) has been widely employed to enhance signal decomposition, helping to separate brain sources as well as neural activity from noise and artifacts [3]. Integrating clinical and behavioral data into ICA frameworks holds promise for uncovering deeper brain-behavior relationships, ultimately advancing personalized approaches to diagnosis and treatment. In this study, we focused on the General Intellectual Ability (GIA) score from the Woodcock-Johnson (WJ) tests because it provides a global index of cognitive performance, integrating multiple domains such as reasoning, memory, and processing speed, which makes it particularly relevant for assessing broad brain-behavior relationships. We hypothesized that incorporating behavioral data, specifically the GIA score, into the ICA decomposition of EEG connectivity data would result in stronger and more stable correlations with cognitive performance than conventional ICA applied to EEG connectivity alone.

ICA has emerged as a powerful statistical technique for modeling covariate effects, particularly in the context of high-dimensional data such as neuroimaging and other biomedical applications. Unlike traditional methods that often rely on univariate approaches, ICA allows for the exploration of multivariate relationships, revealing hidden structures and patterns in complex datasets. ICA is a computational method widely used in EEG signal processing to separate mixed signals recorded from electrodes into their underlying independent sources. EEG data contain a mixture of neural signals, artifacts (e.g., eye blinks, muscle activity), and noise, and ICA helps disentangle these components by assuming that the observed signals are linear mixtures of statistically independent sources. In the context of EEG, these sources can represent different neural processes or artifacts. Delorme et al. demonstrated that ICA is effective in separating brain activity from noise and artifacts [4]. By maximizing the statistical independence of the extracted components, ICA isolates meaningful brain activity patterns while separating artifacts and noise, enabling cleaner and more interpretable data for analysis.

In resting-state EEG studies, ICA has also been used to identify functional connectivity networks. Li et al. applied ICA to resting-state EEG data, revealing how covariates such as mental fatigue and sleep quality affect connectivity patterns [5]. ICA has also been applied in clinical settings to understand the neural correlates of neurological and psychiatric disorders. For instance, Hoffstaedter et al. utilized ICA to investigate the effects of covariates such as medication status and symptom severity in patients with attention-deficit/hyperactivity disorder (ADHD) [6]. ICA is not limited to EEG time series and may also be applied to other types of signals. In functional magnetic resonance imaging (fMRI), the application of component analysis to groups of subjects employs spatial constraints to improve the detection of the default mode network (DMN), a critical component in fMRI studies [7,8]. Further advancements in methodologies are illustrated by Kalyanam et al., who applied ICA to analyze magnetic resonance spectra, demonstrating the versatility of ICA [9]. Additionally, Liu et al. showcased how ICA can effectively reveal functional connectivity networks, underscoring its utility in understanding brain dynamics in various states [10].

Consistent with studies using ICA to decompose new signals that do not necessarily perfectly align with the ICA linearity assumption, we explored a new way to use ICA to analyze EEG data alongside behavioral measures. We applied ICA to brain connectivity data and then checked how the resulting components related to the behavioral measures. We compared standard ICA with including the behavioral measures directly in the data before running ICA, allowing the behavioral and brain data to influence the decomposition process together. The hypothesis we tested was whether the behavioral information integrated into the ICA analysis has the potential to increase the correlation between brain connectivity and behavioral measures.

## Materials and methods

Participants

To investigate the relationship between brain connectivity and behavioral measures, we analyzed 175 patients (mean age 29 with a standard deviation of 16 years; 39 females and 136 males), who presented with a range of conditions, including learning disorders, eating disorders, anxiety, depression, ADHD, and other neuropsychiatric and developmental challenges. All participants possessed at least a high school level of education. The study included all clients who enrolled in the program between January 2022 and December 2024. Inclusion criteria were minimal: participants were required to be at least 18 years old, capable of providing informed consent, and enrolled in the program during the specified time frame. Exclusion criteria were limited to individuals with incomplete data or inability to complete assessments. All records were de-identified to protect the anonymity of individual health information. All participants provided written consent to participate in the study. The study received ethical approval (Pearl Institutional Review Board (IRB) issued approval #20-NEUR-101).

WJ measures

The WJ measures, derived from the Woodcock-Johnson Tests [11,12], are a widely used set of standardized cognitive assessments designed to evaluate a range of intellectual and cognitive abilities. Key domains assessed include verbal ability, working memory, auditory working memory, and GIA, providing valuable insights into linguistic reasoning, short-term information processing, and overall intellectual functioning. These measures are applied in educational, clinical, and research contexts to identify cognitive strengths and weaknesses, diagnose learning disabilities, and explore relationships between cognitive abilities and brain function. In neuroscience, WJ measures are frequently used to study the interplay between cognitive performance and neural connectivity patterns, offering critical data for understanding brain-behavior relationships. Here, we used the most common WJ measures of GIA. All WJ GIA scores were standardized according to the published test norms, resulting in standard scores with a mean of 100 and a standard deviation of 15. No imputation or interpolation was performed, as only participants with complete GIA scores were included in the analysis.

EEG data collection

EEG data were acquired using a 19-channel system conforming to the international 10-20 electrode placement standard (Fp1, F3, C3, P3, O1, F7, T3, T5, Fz, Fp2, F4, C4, P4, O2, F8, T4, T6, Cz, Pz, A2) with reference as the left ear (A1) during a five-minute resting-state condition with eyes closed. Preprocessing was performed using NeuroGuide’s (Applied Neuroscience, Inc., St. Petersburg, FL, USA) automated artifact rejection algorithm with default settings, which apply spectral and statistical thresholds to exclude segments containing ocular, muscular, or technical artifacts. The data were then band-pass filtered from 1 to 30 Hz and re-referenced to linked ears.

EEG connectivity analysis

The connectivity measures used in this study were computed using NeuroGuide [12], a quantitative EEG (qEEG) analysis software, which saved the results in a file named svloretaconnect.txt. This file contains connectivity data derived from the standardized weighted low-resolution electromagnetic tomography (swLORETA) algorithm, a robust method for source localization and functional connectivity analysis. Specifically, we focused on metrics related to the DMN, analyzing connectivity between predefined brain regions. Of all the connectivity measures available, we choose lagged coherence as our EEG connectivity measure because it specifically accounts for time-lagged interactions, which helps to mitigate the confounding effects of volume conduction - a common issue in EEG analysis where signals from nearby sources can create artificial synchrony. Unlike instantaneous coherence, which may overestimate connectivity due to shared volume conduction effects, lagged coherence isolates true neural interactions by focusing on delayed synchronization between regions. This makes it a more reliable indicator of functional connectivity and better suited for capturing meaningful brain dynamics related to cognition. Additionally, compared to phase difference and information flow, which provide insights into phase relationships and directional influences, lagged coherence strikes a balance between robustness to artifacts and sensitivity to genuine neural interactions, making it an optimal choice for our analysis. This metric was calculated across standard EEG frequency bands, including delta, theta, alpha, beta, and gamma, providing a comprehensive view of connectivity dynamics. Only the upper alpha (10-12 Hz) frequency band is included for this specific analysis. 

Independent component analysis (ICA)

In EEG analysis, ICA is a computational technique used to separate mixed signals into statistically independent components, aiding in artifact removal and identifying meaningful neural activity. The EEG signal, represented as a matrix, is assumed to be a linear mixture of independent source signals $S$ through a mixing matrix $A$, such that:

\[ X = A S \]

The goal of ICA is to estimate an unmixing matrix $W$ that separates the observed EEG signals into independent components, expressed as:

\[ S = W X \]

ICA operates under the assumption that the sources are statistically independent and non-Gaussian, except for at most one Gaussian source. We employed Infomax ICA [13], an algorithm that iteratively optimizes to maximize the statistical independence of the extracted components by minimizing mutual information. This approach is widely used in EEG analysis for isolating artifacts such as eye blinks and muscle activity, as well as identifying neurophysiologically meaningful brain sources.

Method 1: ICA on Connectivity Data

In this approach, we first performed ICA to decompose the connectivity data into independent components. Once the independent components were identified, we correlated these components with the WJ measures.

Let $C \in \mathbb{R}^{n \times m}$ denote the connectivity matrix for all subjects, where $n$ is the number of subjects and $m$ represents the number of connections (30 in our case, as described in Appendix A). Each element $C$ corresponds to the connectivity strength between two regions, excluding self-connections.

We then perform ICA to decompose $C$ into $m$ independent components. The decomposition can be represented as: $S = W C$ where $S \in \mathbb{R}^{m \times n}$ is the source matrix and $W \in \mathbb{R}^{m \times m}$ is the weight matrix.

After the ICA decomposition, using new data $\tilde{C}$ from $\tilde{n}$ subjects not used for the ICA decomposition, we compute the ICA source for these subjects $\tilde{S} = W \tilde{C}$ and the correlation between each component and each of the WJ measures: Let $\tilde{J} \in \mathbb{R}^\tilde{n}$ represent the vector of a given WJ measure from the subjects in $\tilde{C}$. We then compute the correlation $\rho$ between each independent component $\tilde{S}_i$ and $\tilde{J}$ across subjects: $\rho(\tilde{S}_i, \tilde{J}) = \frac{\text{Cov}(\tilde{S}_i, \tilde{J})}{\sigma_{\tilde{S}_i} \sigma_\tilde{J}}$ where $\tilde{S}_i \in \mathbb{R}^\tilde{n}$ is the $i$-th independent component vector across $\tilde{n}$ subjects, $\text{Cov}(\tilde{S}_i, \tilde{J})$ is the covariance between $\tilde{S}_i$ and $\tilde{J}$, $\sigma_{\tilde{S}_i}$ and $\sigma_\tilde{J}$ are the standard deviations of $\tilde{S}_i$ and $\tilde{J}$, respectively.

We finally take the max correlation across all components $i$: $\rho_{\text{max}} = \max_{i} \rho(\tilde{S}_i, \tilde{J})$ where $\rho(\tilde{S}_i, \tilde{J})$ is the correlation between the $i$-th independent component $\tilde{S}_i$ and the WJ measure $\tilde{J}$. The statistics of $\rho_{\text{max}}$ across the WJ measures and connectivity measures are assessed.

Method 2: ICA on Connectivity Data and Behavioral Data

In the second approach, we augmented the connectivity data by including the WJ measures before running ICA. By adding the WJ measures as additional features, we allowed the ICA to account for both the brain connectivity and the cognitive variables simultaneously.

Let $C{\prime} \in \mathbb{R}^{n \times (m+1)}$ denote the augmented connectivity matrix, where the WJ measures $J \in \mathbb{R}^n$ are appended as an additional feature to each subject’s connectivity data. The augmented matrix can be represented as $C{\prime} = \begin{bmatrix}C & J\end{bmatrix},$ where $C \in \mathbb{R}^{n \times m}$ is the original connectivity matrix.

We then perform ICA to decompose $C{\prime}$ into $m+1$ independent components. The decomposition is represented as $S{\prime} = W{\prime} C{\prime}$, where $S{\prime} \in \mathbb{R}^{(m+1) \times n}$ is the source matrix, $W{\prime} \in \mathbb{R}^{(m+1) \times (m+1)}$ is the weight matrix.

After the ICA decomposition, using new data $\tilde{C{\prime}}$ from $\tilde{n}$ subjects not used for the ICA decomposition, we compute the ICA source for these subjects $\tilde{S{\prime}} = W \tilde{C{\prime}}$ and the correlation between each component and each of the WJ measures: Let $\tilde{J} \in \mathbb{R}^\tilde{n}$ represent the vector of a given WJ measure from the subjects in $\tilde{C{\prime}}$. We then compute the correlation $\rho$ between each independent component $\tilde{S{\prime}}_i$ and $\tilde{J}$ across subjects: $\rho(\tilde{S{\prime}}_i, \tilde{J}) = \frac{\text{Cov}(\tilde{S{\prime}}_i, \tilde{J})}{\sigma_{\tilde{S{\prime}}_i} \sigma_\tilde{J}}$ where $\tilde{S{\prime}}_i \in \mathbb{R}^\tilde{n}$ is the $i$-th independent component vector across $\tilde{n}$ subjects, $\text{Cov}(\tilde{S{\prime}}_i, \tilde{J})$ is the covariance between $\tilde{S{\prime}}_i$ and $\tilde{J}$, $\sigma_{\tilde{S}_i}$ and $\sigma_\tilde{J}$ are the standard deviations of $\tilde{S{\prime}}_i$ and $\tilde{J}$, respectively.

We finally take the max correlation across all components $i$: $\rho_{\text{max}} = \max_{i} \rho(\tilde{S{\prime}}_i, \tilde{J})$ where $\rho(\tilde{S{\prime}}_i, \tilde{J})$ is the correlation between the $i$-th independent component $\tilde{S{\prime}}_i$ and the WJ measure $\tilde{J}$. The statistics of $\rho_{\text{max}}$ across the WJ measures and connectivity measures are assessed.

This approach incorporates the WJ measures into the feature space of the connectivity data but ensures that only the weights corresponding to the original connectivity features are used when computing correlations with the WJ measures. The statistics of $\rho{\prime}_{\text{max}}$ are then compared to those from Method 1 to evaluate the effects of integrating cognitive variables directly into the ICA framework.

Evaluating method performance

To determine which method was more effective, we computed the best Pearson correlation coefficient (r) between the component activity derived from ICA and the WJ measures across 175 individuals. We employed a jackknife resampling approach, a form of leave-one-out cross-validation, to assess significance. Specifically, in each of the 175 iterations, we used data from 174 individuals as the training set to compute the Pearson correlation, while the remaining individual served as the testing set to evaluate the generalizability of the result. This process was repeated systematically, omitting a different individual in each iteration. By doing so, we obtained a distribution of Pearson r values, allowing us to estimate the stability and significance of the observed associations between brain connectivity and cognitive measures.

To determine whether one method was preferable to the other, we conducted a pairwise comparison of p-values obtained for each approach. First, we applied a parametric t-test, which assumes normality in the distribution of differences, to evaluate whether the observed differences were statistically significant. Since the distribution was not Gaussian, we then performed a surrogate permutation test, which involved randomly shuffling the data to create a null distribution, allowing us to assess whether the observed differences were greater than what would be expected by chance [14].

## Results

**Figure 1:**
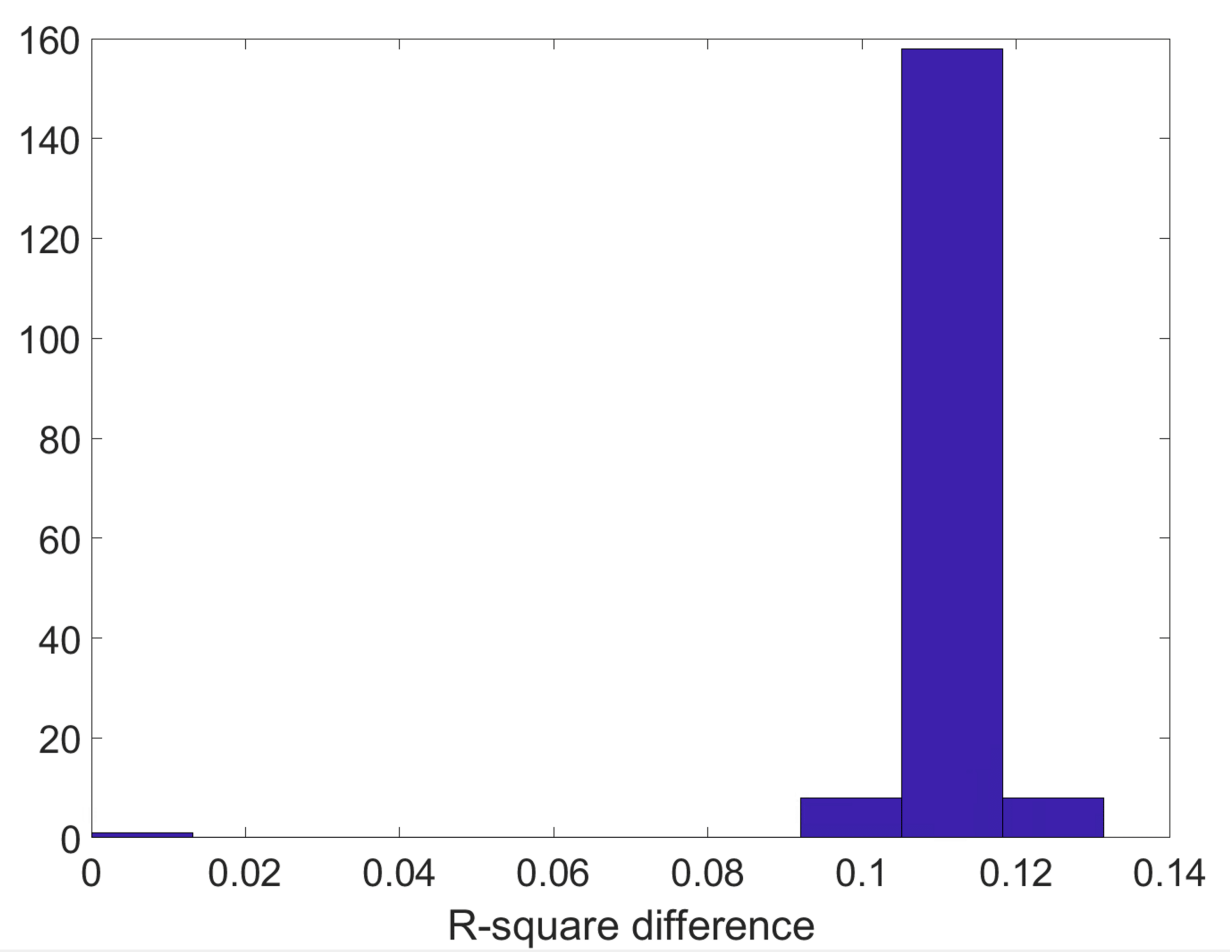
Comparison of ICA R-square results with and without incorporating the WJ questionnaire data. Histogram of differences in Pearson correlation R-square values, where positive values indicate stronger R-square values when the questionnaire data is included. ICA: independent component analysis; WJ: Woodcock-Johnson

**Figure 2:**
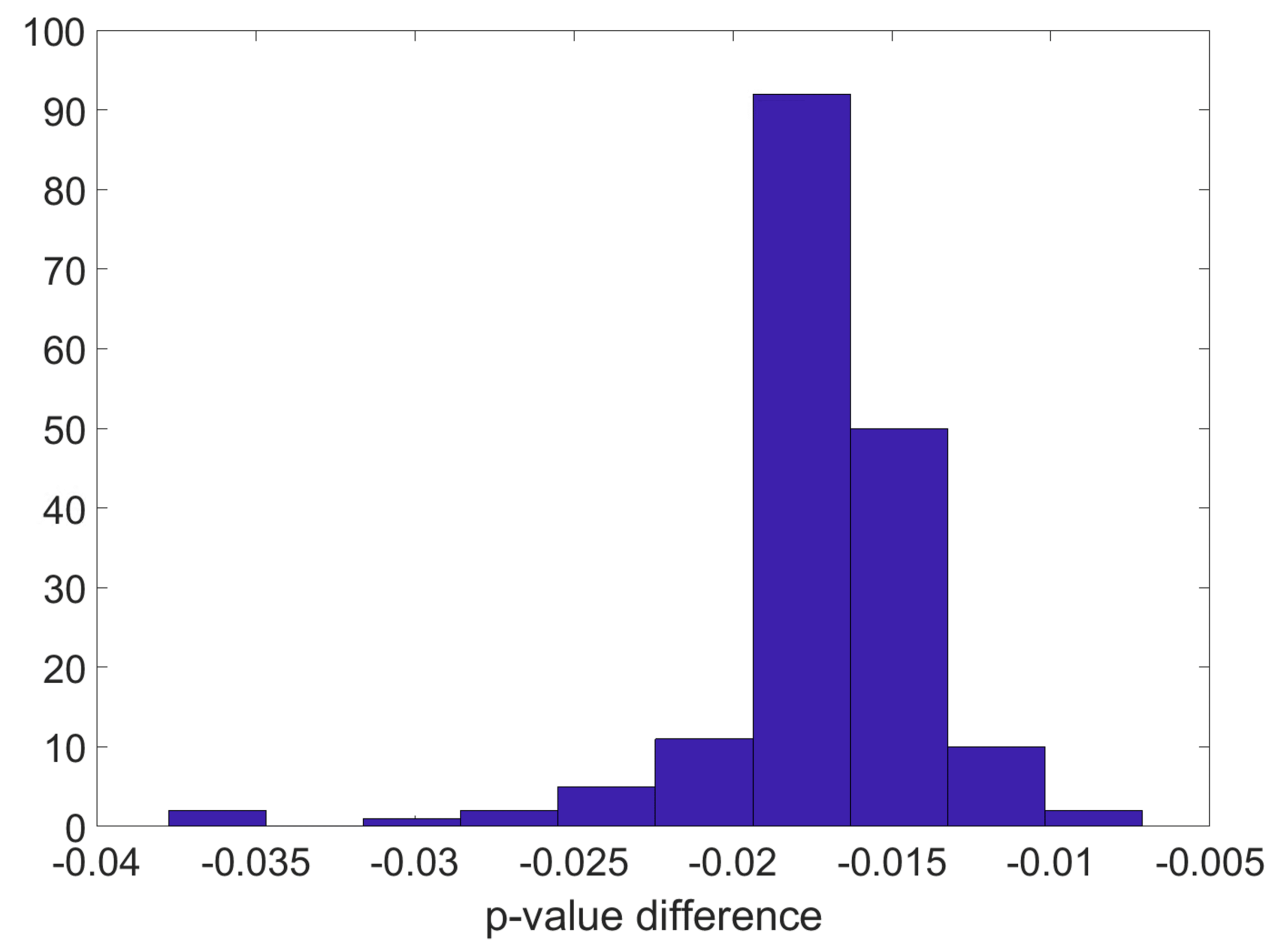
Comparison of ICA p-value results with and without incorporating the WJ questionnaire data. Histogram of differences in Pearson correlation p-values, where negative values indicate stronger statistical significance when the questionnaire data is included. This analysis shows that the differences are systematically biased, with more negative than positive values. ICA: independent component analysis; WJ: Woodcock-Johnson

Figures 1-2 show the distribution of the differences in R-squared and the corresponding p-values when comparing the ICA results obtained with and without incorporating the WJ questionnaire data into the analysis of the test data (see Materials and Methods section). Negative values on the X-axis indicate that the ICA with the questionnaire yielded lower p-values (i.e., stronger statistical significance) compared to the ICA without the questionnaire. However, a parametric paired t-test was significant (p=0.01, degree of freedom of 174). The distribution is not Gaussian (a Kolmogorov-Smirnov test of Gaussianity fails and indicates a non-Gaussian distribution with p<10^-10^), so we used a permutation surrogate test. This permutation test returns p<0.0007, indicating that the p-values for the ICA results incorporating the questionnaire are more significant than those that do not include it.

## Discussion

This study demonstrates that integrating cognitive performance metrics from the WJ tests into ICA of EEG connectivity data enhances the statistical significance and explained variance of brain-behavior relationships. Compared to conventional ICA, the inclusion of behavioral measures resulted in stronger correlations between EEG components and cognitive performance on the test set, suggesting that this approach captures more meaningful neural patterns. Clinically, these findings highlight the potential of ICA with integrated covariates as a valuable tool for identifying neurophysiological markers of cognitive function. This method could improve the assessment of cognitive deficits in neuropsychiatric and developmental disorders, paving the way for more personalized and targeted interventions.

Shi and Guo introduced a framework for modeling covariate effects in group ICA (GICA) with applications in fMRI, demonstrating how covariate integration can refine the extraction of meaningful brain networks [15]. Our study builds on this approach by extending ICA decomposition beyond traditional neuroimaging modalities, incorporating behavioral measures into EEG connectivity analysis. While Shi and Guo emphasized the role of covariates in group-level ICA to control for variability across subjects, we applied a similar principle to individual-level ICA decomposition, directly integrating the GIA cognitive performance metric from the WJ tests. This methodological shift aligns with the broader goal of enhancing the interpretability of ICA-derived components, particularly in clinical neuroscience, where behavioral assessments offer crucial insight into cognitive function. Our results reinforce the potential of ICA as a powerful multivariate framework for uncovering brain-behavior relationships, echoing Shi and Guo’s emphasis on the importance of incorporating relevant covariates to enhance component interpretability.

While our findings demonstrate improved statistical significance and explained variance through the integration of behavioral covariates into ICA, several limitations must be acknowledged. First, our cohort consisted of a heterogeneous clinical population, and subgroup analyses were not performed. It is therefore possible that variability in clinical profiles influenced the observed associations. Second, we focused exclusively on the GIA score as a behavioral covariate due to its broad integration of multiple cognitive domains, but future studies may benefit from examining the influence of other cognitive subscores to refine brain-behavior mappings. Finally, while the observed statistical improvements suggest stronger associations, the clinical relevance of these enhanced correlations remains to be established. Future work will be needed to evaluate whether the improved associations translate into practical benefits for diagnosis, prognosis, or treatment planning.

Although we used a training and testing set, one limitation of this study is the lack of cross-validation in the training process, which could be implemented to further assess the stability and generalizability of our findings. While our approach focused on comparing ICA with and without the inclusion of the variable of interest (VOI), incorporating cross-validation would allow us to systematically test the robustness of the results by running multiple iterations with slightly different training data. This would help confirm whether the observed improvements in statistical significance and explained variance hold consistently across different subsets of data. Additionally, given the potential issue of multiple comparisons when analyzing all cognitive measures and networks, cross-validation could assist in refining the selection process by identifying the most reliable and relevant associations. However, for this study, we mitigated this concern by preselecting one VOI, ensuring a focused comparison that clearly demonstrated the advantage of integrating cognitive measures into ICA. Future work could extend this approach by employing cross-validation across multiple VOIs and networks, enhancing the reliability and applicability of ICA in clinical and cognitive neuroscience.

Future research could also benefit from comparing the proposed ICA method with other multivariate techniques such as Partial Least Squares Regression (PLSR) and Canonical Correlation Analysis (CCA). PLSR is particularly useful for identifying features that maximize the covariance between neural connectivity patterns and cognitive performance measures, allowing for the selection of the most relevant brain-behavior associations [16]. Similarly, CCA provides a powerful framework for uncovering linear relationships between two sets of variables, such as EEG connectivity features and cognitive test scores, by maximizing their shared variance [16]. Comparing ICA with these methods would help determine whether incorporating behavioral measures directly into the ICA decomposition offers unique advantages over more traditional regression-based approaches. Future studies should evaluate whether ICA with integrated covariates provides superior sensitivity and interpretability compared to PLSR and CCA, ensuring that the most effective multivariate techniques are used for studying brain-behavior relationships in clinical neuroscience.

While the observed improvements in statistical significance suggest stronger brain-behavior associations, the clinical implications remain to be established. Whether these enhanced correlations are sufficient to inform diagnosis, prognosis, or treatment decisions requires further investigation in larger and independent cohorts. Future studies incorporating external validation will be essential to assess the practical utility of this approach.

## Conclusions

In conclusion, this study demonstrates that integrating cognitive performance metrics into ICA enhances the detection of meaningful brain-behavior relationships in EEG connectivity data. By incorporating behavioral covariates, we observed improved statistical significance and explained variance, suggesting that this approach captures more robust neural patterns associated with cognitive function. These findings highlight the potential of ICA with integrated covariates as a valuable tool in clinical neuroscience, offering a more comprehensive framework for studying cognitive deficits in neuropsychiatric and developmental disorders.

## Appendices

Appendix A

**Table 1:** Pairs of brain areas in the Talairach space Pairs of brain regions in Talairach space used to compute connectivity measures within the default mode network in the upper alpha (10-12 Hz) frequency band, as defined by NeuroGuide. The numbers refer to Talairach area indices, with “L” and “R” denoting the left and right hemispheres, respectively.

Origin	Destination
10L	23L
10R	23R
10L	31L
10R	31R
7L	10L
7R	10R
10L	39L
10R	39R
10L	40L
10R	40R
10L	32L
10R	32R
23L	31L
23R	31R
7L	23L
7R	23R
23L	39L
23R	39R
23L	40L
23R	40R
7L	31L
7R	31R
31L	39L
31R	39R
31L	40L
31R	40R
7L	39L
7R	39R
7L	40L
7R	40R
